# Magnesium: Are We Consuming Enough?

**DOI:** 10.3390/nu10121863

**Published:** 2018-12-02

**Authors:** Mohammed S. Razzaque

**Affiliations:** 1 Department of Pathology, Lake Erie College of Osteopathic Medicine, Erie, PA 16509, USA; mrazzaque@lecom.edu; Tel.: +1-814-860-5127; 2 Department of Oral Health Policy & Epidemiology, Harvard School of Dental Medicine, Boston, MA 02115, USA; 3 Department of Preventive & Community Dentistry, School of Dentistry, College of Medicine & Health Sciences, University of Rwanda, Kigali, Rwanda; 4 College of Advancing & Professional Studies (CAPS), University of Massachusetts Boston (UMB), Boston, MA 02125, USA

**Keywords:** vitamin D, magnesium, bone, deficiency

## Abstract

Magnesium is essential for maintaining normal cellular and organ function. In-adequate magnesium balance is associated with various disorders, such as skeletal deformities, cardiovascular diseases, and metabolic syndrome. Unfortunately, routinely measured serum magnesium levels do not always reflect total body magnesium status. Thus, normal blood magnesium levels eclipse the wide-spread magnesium deficiency. Other magnesium measuring methods, including the magnesium loading test, may provide more accurate reflections of total body magnesium status and thus improve identification of magnesium-deficient individuals, and prevent magnesium deficiency related complications.

## 1. Introduction

Nutrients act in a coordinated manner to maintain physiologic cellular and tissue functions, and thus, their dysregulation could adversely affect organ functions [1,2,3,4,5,6,7,8,9]. Magnesium is the fourth most abundant mineral in the human body. This nutrient serves as a cofactor or activator in more than 600 enzymes and influences extracellular calcium levels [10]. Magnesium is indispensable for maintaining normal cellular functions, as it is utilized in RNA and DNA syntheses, antioxidant level maintenance in the cell and energy metabolism [11,12,13]. Magnesium in the human body is primarily located in the intracellular space (40%) or in bones and teeth (60%) [13,14,15,16].

Of clinical importance, around 0.3% of total body magnesium is found in serum. Thus, total and/or ionized magnesium concentrations measured in plasma or serum are not reliable markers of total magnesium levels in the body; as serum magnesium does not reflect the total magnesium content at the tissue or organs, and is also a poor indicator of intracellular magnesium content (Box 1) [16,17]. A 1974 study determined that a normal serum magnesium reference value is considered to be between 1.82 to 2.30 mg/dL (0.75–0.95 mmol/L) [18]. These reference intervals were selected based on the distribution patterns of serum magnesium in a normal population.

Emerging evidence suggests that the serum magnesium/calcium quotient (0.4 is optimal, 0.36–0.28 too low) is a more practical and sensitive indicator of magnesium status and/or turnover, than the serum magnesium level alone [19]. In chronic latent magnesium deficiency, magnesium levels in the blood are within a normal range, despite there being severely depleted magnesium content in the tissues and bones. Therefore, using magnesium levels in the blood to determine total magnesium levels in the body can result in underestimation of magnesium deficiency in healthy and diseased populations. Recent studies have shown that individuals with serum magnesium levels around 1.82 mg/dL (0.75 mmol/L) are most likely to have a magnesium deficiency, while those with serum magnesium level more than 2.07 mg/dL (0.85 mmol/L) are most likely to have adequate levels [20,21]. Of relevance, individuals with serum magnesium levels between the 0.75 to 0.85 mmol/L should be tested with additional measurements to confirm body magnesium status.

Box 1 **What is the clinical question?**

▪ Does serum magnesium level reflect total body magnesium status?

**What does the evidence say?**

▪ Normal level of serum magnesium does not rule out moderate to severe magnesium deficiency.

**What is the take-home message for clinicians?**

▪ Normal level of serum magnesium does not rule out moderate to severe magnesium deficiency.

In humans, red blood cell (RBC) magnesium levels often provide a better reflection of body magnesium status than blood magnesium levels. When the magnesium concentration in the blood is low, magnesium is pulled out from the cells to maintain blood magnesium levels within normal range. Therefore, in case of magnesium deficiency, a blood test of magnesium might show normal levels, while an RBC magnesium test would provide a more accurate reflection of magnesium status of the body. For exact estimation of RBC magnesium level, individuals are advised not to consume vitamins, or mineral supplements for at least one week before collection of RBC samples. A normal RBC magnesium level ranges between 4.2 and 6.8 mg/dL. However, some experts recommend aiming for a minimum level of 6.0 mg/dL on the RBC test. While not commonly available, some places use the Non-invasive Intracellular Mineral-Electrolyte Analysis (EXA) test to determine tissue levels of magnesium. Some laboratories prefer to use a hair mineral analysis test, which not only reveals mineral deficiencies but heavy metal toxicity. Such tests provide information on the individual mineral levels and their ratio to other minerals in the tissue. Additionally, the composition of minerals deposited in the hair could be reflective of overall body chemistry and health status.

Clinical diagnosis of magnesium deficiency is not simple, as symptoms associated with magnesium deficiency are unspecific, and generally confounded by low consumption of other nutrients. Intravenous or oral magnesium loading tests used in combination with magnesium excretion concentrations from a 24-h urine specimen may be more useful for detecting subclinical magnesium deficiency. Studies have shown that, in healthy subjects, intravenous magnesium load retention is around 2–8% [22,23]. Load retention >27%, following an intravenous magnesium load is considered highly indicative of magnesium deficiency [22,24]. It needs to be mentioned, however, that intravenous or oral magnesium loading tests require normal kidney or gastrointestinal functions to get an accurate reflection of magnesium status. Various available methods for the measurements of magnesium are listed in Table 1.

## 2. Sources of Magnesium

The daily recommended intake of magnesium for the adults is around 300–400 mg/day. The rate of intestinal absorption of dietary magnesium depends on the amount consumed and total body magnesium status; such intestinal uptake occurs by both active and passive absorption. Active transcellular magnesium uptake is achieved by magnesium channels in the large intestine, including Transient Receptor Potential Melastin (TRPM) 6 and TRPM 7. The electrochemical gradient facilitates passive magnesium absorption, mostly occurring in the small intestine.

Magnesium homeostasis is maintained by renal reabsorption and urinary excretion [25]. In the case of a magnesium surplus, renal excretion increases while in deficiency, renal uptake of magnesium increases to minimize the loss. Despite such renal conservation, magnesium is also pulled from its skeletal storage to keep serum levels within the normal range, which predisposes an individual to osteopenia, osteoporosis or fracture, regardless of normal serum magnesium levels. When magnesium consumption is <250 mg/day, around 40–80 mg magnesium is excreted per day; excretion rises to 80–160 mg/day when consumption is >250 mg/day [20]. Of relevance, urinary magnesium excretion does not change immediately after consumption, rather it takes a few days. Therefore, a single 24-h urinary magnesium estimation might not provide exact magnesium status, and the possibility exists that urinary magnesium excretion may be low or high despite normal magnesium excretion [20].

Many naturally grown foods contain magnesium, but its consumption has significantly decreased in the last few decades because of changes in dietary habits; furthermore, removal of magnesium during food processing also contributes to reduced magnesium uptake. Foods high in magnesium include almonds, bananas, black beans, broccoli, brown rice, cashews, flaxseed, green vegetables (spinach), nuts, oatmeal, seeds (pumpkin, sesame, sunflowers) soybeans, sweet corn, tofu, and whole grains.

As magnesium plays an important role in a wide-range of cellular functions, from maintaining ionic gradients to mitochondrial oxidative phosphorylation to DNA/RNA synthesis to cellular signaling, it is not unexpected to find that magnesium deficiency results in various systemic diseases. Analysis of a cohort of 286,668 healthy individuals and 10,192 type II diabetes patients revealed that an inverse association was found between magnesium intake and the incidence of type II diabetes; from the findings of this meta-analysis, the authors recommended that increased consumption of magnesium-rich foods may reduce the risk of type II diabetes [26]. Similar observations were noted in Canadian Health Measures Survey (cycle 3, 2012–2013); type I and type II diabetes were associated with lower serum magnesium level (0.04–0.07 mmol/L) when compared to individuals without diabetes. In addition, body mass index, glycated hemoglobin, serum glucose and insulin concentrations, and homeostatic model assessment of insulin resistance were negatively associated with serum magnesium level [27].

Sodium-glucose cotransporter 2 (SGLT2) inhibitors are clinically used for the treatment of type II diabetes. SGLT2 inhibitors selectively inhibit renal glucose reabsorption and increase urinary glucose excretion to lower glucose levels. Analysis of data collected from 15,309 patients showed significantly higher magnesium serum magnesium levels in SGLT2 inhibitor-treated patients, compared to untreated patients. Further studies are needed to determine whether the benefits of SGLT2 inhibitor treatment in diabetes patients are partly achieved through altered magnesium homeostasis induced by SGLT2 inhibitor [28].

In addition to increasing dietary magnesium intake, in certain disease conditions, exogenous magnesium supplementation might be needed, either taken orally or topically using magnesium oil. Various forms of magnesium supplements are available, including magnesium citrate, magnesium glycinate, magnesium threonate and magnesium malate. Different magnesium preparations have different intestinal absorption ability. Studies have claimed that Organic magnesium supplements (asparate, citrate, lactate, chloride) have been shown to be more bioavailable than those with inorganic magnesium (oxide, sulfate) [29]. This is still a developing area of research, as there are studies that reported no substantial differences among these formulations, in regard to bioavailability [30].

A common unpleasant side effect of oral magnesium supplementation is diarrhea. By applying magnesium oil on to the skin, one could minimize such undesirable side effects of oral magnesium preparations. Recent studies have claimed that transdermal magnesium is absorbed through sweat glands [19]. Of relevance, topical application of a cream containing 2% magnesium reduced diaper dermatitis and diaper rash in children [31]. Epsom salt (magnesium sulfate) baths are used as a home remedy against abdominal pain, constipation, and muscle strains. Epsom salt is also believed to enhance magnesium status. However, high ingestion of Epsom salt may result in undesirable complications [32]. Although hypermagnesemia is not common in clinical practice, it can induce hypotension, bradycardia and in extreme situations coma. Hypermagnesemia is usually related to the administration of high doses of magnesium, magnesium-containing drugs, or kidney diseases.

## 3. Magnesium Deficiency

Magnesium deficiency is commonly the result of reduced consumption or inadequate absorption and/or increased excretion from the body. A wide range of human diseases, including cardiovascular and metabolic diseases, skeletal disorders, respiratory illness and neurologic anomalies (stress, depression, and anxiety) are linked to magnesium inadequacy. Magnesium is an important constituent of bone and plays a vital role in bone mineralization, partly by influencing synthesis of active vitamin D metabolites [33,34], which support intestinal calcium and phosphate absorption [35,36]. Studies have shown that hypovitaminosis D-associated risk of mortality could be reduced by the consumption of magnesium [12,37,38,39]. According to the National Health and Nutrition Examination Survey (NHANES) data, higher uptake of magnesium reduced the risk of vitamin D deficiency and/or insufficiency in the general population [40]. Thus, vitamin D supplementation could be reduced by consuming an adequate amount of magnesium, as magnesium helps in the activation or stimulation of endogenous vitamin D already present in the body to exert its functions [41]. This is beneficial for individuals dependent on exogenous vitamin D supplements as they are not always risk-free, particularly when consumed in higher doses or used for prolonged periods [42]. Of clinical importance, some symptoms of excessive vitamin D consumption are similar to magnesium deficiency. Muscle spasms and cramps are usually related to high calcium levels relative to the magnesium levels, and in such situations, the dose of vitamin D supplement dosage may need to be readjusted.

A positive association between dietary magnesium intake and BMD is also reported [43,44]. A study of osteoporotic postmenopausal women who consumed oral magnesium citrate for 30 days revealed resulting biochemical features of suppressed bone turnover [45]. It is speculated that widely available environmental factor fluoride can bind with magnesium to generate magnesium-fluoride or sellaite. This complex can replace magnesium that is present in the bone and cartilage, to make the skeletal system more prone to fracture.

In addition, optimal magnesium level is essential for normal cardiac functions by stabilizing the rhythm of the heart. This nutrient also plays a role in averting abnormal cardiovascular clotting. Magnesium is believed to contribute to blood pressure maintenance as well [46,47,48]. In a meta-analysis with 20,119 cases of hypertension, an inverse association was found between dietary magnesium uptake and hypertension. Such an association was noted between individuals consuming a high level of magnesium (>300 mg) thus consuming a low level of magnesium (<200 mg) [49]. Individuals taking medication should consult their physician to ensure the medication is not causing magnesium wasting, as many high blood pressure drugs or diuretics can increase magnesium removal from the body.

Studies found that magnesium is effective in reducing the rate of cardiac attacks and cerebrovascular strokes [12,50,51]. A meta-analysis of 532,979 individuals revealed an inverse association between dietary magnesium uptake and risk of cardiovascular events (coronary heart disease, stroke) [52]. Of particular clinical importance, the highest risk reduction occurred when magnesium intake was increased from 150 mg to 400 mg. To achieve and maintain a healthy magnesium status, in addition to consuming natural foods and vegetables high in magnesium measures should be taken to reduce the loss of magnesium from the body by limiting the consumption of coffee and soda beverages, reducing salt and sugar uptake, and by avoiding alcohol consumption.

## 4. Conclusions

**Figure 1:**
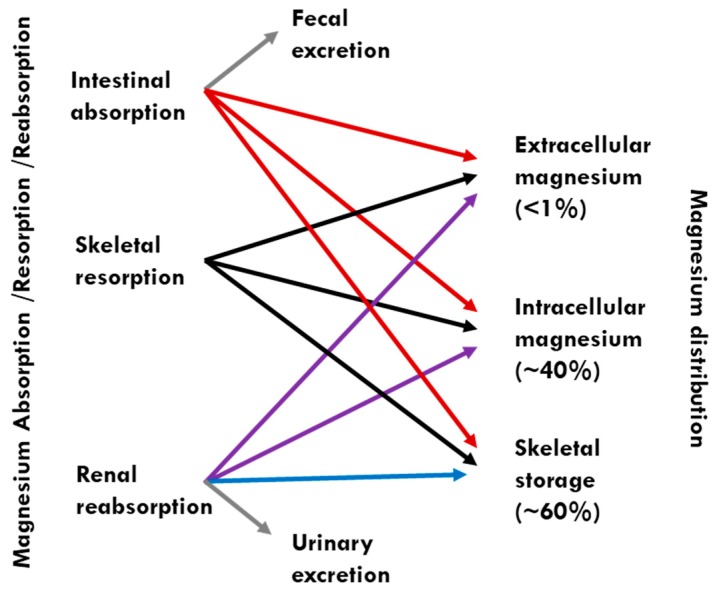
Total body magnesium homeostasis is primarily maintained by the multi-organ cross-talk among the intestines, kidney, and bones. Because less than 1% of total body magnesium is present in serum, serum magnesium concentration does not truly reflect total body magnesium content, or intracellular magnesium content. Despite magnesium deficiency, magnesium level in many organs may remain stable due to effective mobilization of magnesium from the bone deposit, and uptake by the organs [54].

Magnesium deficiency can induce a wide range of clinical complications, including painful muscle spasms, fibromyalgia, arrhythmia, osteoporosis and migraines. A meta-analysis of randomized controlled trials with migraine patients receiving intravenous magnesium (948 participants) or oral magnesium (789 participants) resulted in reduced acute migraine attacks with less frequency and intensity of migraine [53]. Orally consumed magnesium is partly absorbed in the intestine (primarily in the small intestines) and stored in the bone as a mineral. Excessive magnesium is generally excreted through the kidneys (Figure 1) [12,46,54].

Today’s soil is depleted of minerals, and therefore the crops and vegetables grown in that soil are not as mineral-rich as they used to be. Approximately half of the US population consumes less than the required amount of magnesium. Even those who strive for better nutrition in whole foods can fall short, due to magnesium removal during food processing. Improved outreach and education may help reduce wide-spread magnesium deficiency and its related complications to maintain good health. It should be emphasized that vitamin D can positively influence magnesium absorption and support vitamin D metabolism.

This brief article highlights why routinely measured serum magnesium levels do not reflect total body magnesium status resulting in underreporting of the prevalence of magnesium deficiency. As listed in Table 1, other magnesium measuring methods, including the magnesium loading test, may improve identification of magnesium-deficient individuals. Health care providers should take the initiative to increase awareness of magnesium deficiency and encourage the general population to consume magnesium-containing foods to reduce disease burden [55,56,57].

## Figures and Tables

**Table 1:** A partial list of magnesium measurement methods used to determine magnesium status. Please note that serum magnesium level is still the most widely practiced method of determining magnesium status in health and disease [19,20,21,22,23,24].

▪ Total serum magnesium
▪ Oral magnesium loading test
▪ Intravenous magnesium loading test
▪ RBC magnesium content
▪ Hair magnesium content
▪ Muscle magnesium content (biopsy)
▪ Bone magnesium content
▪ 24-h urinary magnesium
▪ The ratio of ionized to total magnesium

RBC, red blood cell.
